# Gender gap in deep brain stimulation for Parkinson’s disease: preliminary results of a retrospective study

**DOI:** 10.1007/s10143-024-02290-7

**Published:** 2024-01-24

**Authors:** Teresa Somma, Ilaria Bove, Francesca Vitulli, Domenico Solari, Andrea Bocchino, Carmela Palmiero, Maria Rosaria Scala, Cesare Zoia, Paolo Cappabianca, Felice Esposito

**Affiliations:** 1 Department of Neurological Sciences, Division of Neurosurgery, Università Degli Studi Di Napoli Federico II, Naples, Italy; 2 UOC Neurochirurgia, Ospedale Moriggia Pelascini, Gravedona Ed Uniti, Italy

**Keywords:** Gender gap, Deep brain stimulation, Surgery, Parkinson’s disease

## Abstract

Subthalamic nucleus deep brain stimulation (STN-DBS) is an effective treatment of PD for both women and men. However, discussions have been reported about the impact of STN-DBS surgery in PD. The aim of our study is to identify differences between men and women in terms of pre- and post-DBS symptoms and try to explain the possible causes. In the current study, we evaluated the gender impact on STN-DBS in PD at the Department of Neurosurgery of University of Naples “Federico II” from 2013 to 2021. Motor and non-motor symptoms were evaluated. To compare the data before and after surgery and between the genders, Wilcoxon-Mann–Whitney tests were performed. A total of 43 patients with PD were included; of them, 17 (39%) were female. Baseline evaluation revealed no gender differences in the age of onset (*p* = 0.87). Not significant differences were noted in the Unified Parkinson’s Disease Rating Scale (UPDRS) pre-surgery score, but if we consider UPDRS subscores of motor examination, significant clinical improvement was reported in both male and female in terms of UPDRS pre- and post-surgery (*p* < 0.001). STN-DBS is a highly effective treatment for motor and non-motor symptoms of PD for both women and men but our study hints towards gender-specific outcomes in motor domains. Improving our knowledge in this field can allow us to implement strategies to identify new directions in the development of an adequate treatment of PD in terms of surgical intervention and in consideration of the gender.

## Introduction

Parkinson’s disease (PD) is the second most common, age-related neurodegenerative disorder, ranging from 3 to 5% in the 65–85-year population [1]. Epidemiological data have demonstrated that men have a greater susceptibility and different onset to PD compared to women [2]. Female patients tend to have milder symptoms in the early stages of the disease, but they have higher risk of developing levodopa-related motor complications (i.e., fluctuations and dyskinesia) [3–5]. Gender differences have also been found in relation to non-motor symptoms in PD patients [6]. The possible mechanisms underlying these differences in PD may involve hormonal factors, genetic predisposition, lifestyle exposure, and functional dopamine pathways.

The role of estrogens in PD remains unclear; differences in circulating estrogen levels between men and women could explain differences in risk of developing the disease, severity of motor symptoms, and motor complications treatment related. Di Luca et al. [7, 8] tried to investigate whether there were racial, ethnic, or gender differences, in terms of access to care in PD, stating how women and patients those with lower income were less likely to be referred to access to cure. Therefore, biological sex differs from the term “gender” in consideration also of sociocultural aspects.

Hariz et al. [9], in their surgical series of 38 patients who underwent STN-DBS, concluded that women are probably selected less frequently and later than necessary for surgical treatment. Over time, there have been several attempts to understand how gender impact affects the outcome of deep brain stimulation of subthalamic nucleus surgery (STN-DBS) in PD. Several studies have suggested similar improvements of motor and non-motor symptoms after STN-DBS, while others underline a different outcome [7]. Furthermore, previous data have shown disparities in access to DBS between men and women as women are less likely to undergo DBS. Little is known about gender-related differences in post-surgical outcomes or distinct nonmotor and motor profiles that could explain the “gender gap.” Nowadays, studies considering female gender as variable of different outcome are highly under-represented especially in PD research. Despite the fact that gender differences about the epidemiological and clinical features in PD are reported widely, the gender impact of DBS in terms of post-operative outcomes is not clear. The aim of our study is to identify differences between men and women in terms of pre- and post-DBS symptoms and try to explain the possible causes.

## Materials and methods

We evaluated patients with PD who underwent bilateral STN DBS for PD enrolled from 2013 to 2021 at the Department of Neurosurgery of University of Naples “Federico II.” Patients underwent to the bilateral implantation of quadripolar DBS electrodes (Medtronic, MN, USA) with a selective targeting on the dorso-lateral region of the subthalamic nucleus (STN) according to the standard protocol described in literature [10–12]. All procedures performed in studies involving human participants were in accordance with the ethical standards of the institutional and/or national research committee and with the 1964 Helsinki declaration and its later amendments or comparable ethical standards. For this type of study, formal consent was required. All patients were evaluated preoperatively by a multidisciplinary team of neurologists, psychiatrists, and neurosurgeons who assessed the eligibility for surgery by the administration of the core assessment program for surgical interventional therapies in Parkinson’s disease (CAPSIT-PD) and MDS-PD criteria [13, 14] (Table 1). The Unified Parkinson’s Disease Rating Scale (UPDRS) was applied to characterize non-motor and motor aspects (UPDRS I and II) and motor complications (UPDRS IV) of the disease [9]. UPDRS part III consists of the standardized motor examination. All patients were assessed using UPDRS I–IV 30 days before and 1 year after the DBS. The UPDRS subscores of motor and non-motor, cognitive impairment (ITEM 1.1-UPDRS I), non-motor and motor aspects of experiences of daily living (nM-EDL; M-EDL-UPDRS II), and motor complications (UPDRS IV) were examined for all patients. The UPDRS III motor examination assessment was performed in both OFF-medication condition (MedOF) after a 12-hwithdrawal from drugs, and in the ON-medication condition (MedON). The UPDRS III motor examination assessment was performed in both OFF-medication condition (MedOF) after a 12-h withdrawal from drugs, and in the ON-medication condition (MedON).

**Table 1:** Core assessment program for surgical interventional therapies in Parkinson’s disease CAPSIT-PD

General and mood evaluation	Mattis Dementia Rating Scale	MDRS
	Montgomery and Asberg Depression Rating Scale	MADRS
Executive function	Verbal fluency: letters F, A, and S	FAS
	Paced Auditory Serial Addition Test	PASAT
	Odd Man Out	OMO
	Modified Brown Peterson Paradigm	MBPP
Explicit memory	Rey Auditory and Verbal Learning Test	RAVLT
	Visual amnesic battery of Signoret	
Procedural memory	Short version of Tower of Hanoi	

### Statistical analysis

The raw data were entered into Microsoft Excel (version 10.14 for Mac). Statistical analyses were done via R (version 4.0.2; The R Foundation for Statistical Computing) and RStudio (version 1.2.1335). Standard descriptive statistics were used to describe the characteristics of cases (median with range, mean ± SD, and frequencies with percentages). Shapiro–Wilk test was used to assess the normality.

A *p*-value < 0.05 was considered statistically significant.

## Results

A total of 43 patients (26 male and 17 women) with PD were included in this study.

Shapiro–Wilk test was performed and showed that the distribution of age at diagnosis, preoperative UPDRS score MedOF/MedON and subscore departed significantly from normality.

**Fig. 1:**
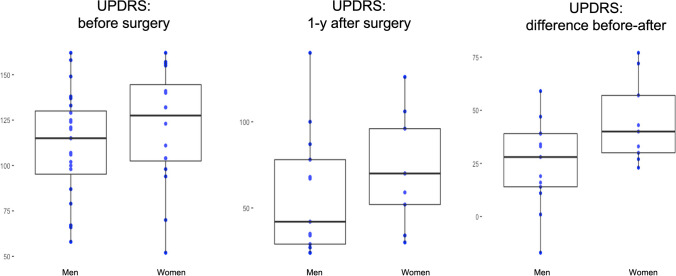
UPDRS boxplot before surgery (left) and after surgery (central) and difference between before and after surgery (right) in men and women

Baseline evaluation revealed no gender differences in the age of onset (*p* = 0.87) (median 50, Min 17, Max 59; 1st Qu 42.5 3rd Qu 52 for men; median 43, Min 28, Max 60; 1st Qu 41, 3rd Qu 56 for women) (Table 2) Preoperatively, no significant differences were noted in the UPDRS score on both MedOF (*p* = 0.98) and MedON (*p* = 0.99) between man and women. If we look to the UPDRS subscores of motor examination, there are no differences in MedON (*p* = 0.68) and MedOF phase (*p* = 0.27), with a significant motor impairment score in women than men (median 34 MedON and 34 MedOF for men; 37 MedON and 47 MedOF for women) (Fig. 1). No significant differences were found in the UPDRS I cognitive functions (*p* = 0.59), in the activities of daily living (ADL) scores (UPDRS II) (*p* = 0.07), and UPDRS IV dyskinesia and fluctuations scores (*p* = 0.76) (Table 2).

**Table 2:** Baseline characteristics of PD patients. *Onset* age at diagnosis; *UPDRS* Unified PD Rating Scale; *ON* on medication; *OFF* off medication; Median; *Qu* quartile

Pre-OP	Men Median, 1st Qu, 3rd Qu	Women Median, 1st Qu, 3rd Qu	*p*-value
Number of patients	26 *n* (61%)	17 *n* (39%)	0.92
Onset	50; 42.5; 52	43; 41; 56	0.87
Age at DSB	60; 54.5; 59.4	59; 52;66.5	**0.04**
UPDRS pre-surgery ON	7.8; 65.5; 88.50	7.8; 6.5, 8.7	0.99
UPDRS pre-surgery OFF	89;77.5;106	8.9; 7.7; 101	0.98
UPDRS I	0,65; 0; 2.55	0.50; 0; 2.27	0.59
UPDRS II	41,90; 34,12; 49,68	39; 30,64; 47,80	0.07
UPDRS III ON	34; 29; 40	3.7; 3.4; 40	0.68
UPDRS III OFF	34; 42; 46	47; 30; 56	0.27
UPDRS IV	12; 8.5; 13.5	12; 8; 12	0.76

Bold represents significant result (*p*<0.05)

At 1-year of follow-up, a statistical significant improvement was found in the UPDRS (I–IV) score in both man (*p* < 0.001) and women (*p* < 0.001) (Table 3). Health-related disability as measured by the ADL as well as non-motor symptoms as measured by the UPDRS II and motor impairment as measured by the UPDRS III score and motor complication as measured by the UPDRS IV did not show any difference in terms of improvement degree when comparing men and women (Table 4).

**Table 3:** Clinical improvement analyzing UPDRS score before and after DBS surgery in men and women. Median; *Qu* quartile

Post-OP	UPDRS pre Median, 1st Qu, 3rd Qu	UPDRS post Median, 1st Qu, 3rd Qu	*p*-value
Men	89; 77.5; 106	42; 24; 62	< 0.001
Women	8.9; 7.7; 101	36; 32; 49	< 0.001

**Table 4:** Post-DBS outcomes. *UPDRS* Unified PD Rating Scale; *EDL* experiences of daily living. Median; *Qu* quartile

Post-OP	Men Median, 1st Qu, 3rd Qu	Women Median, 1st Qu, 3rd Qu	*p*-value
UPDRS	42; 24; 62	36; 32; 49	0.77
UPDRS I	0.73; 0; 2.47	0.56; 0; 2.82	0.74
UPDRS II	30.50; 22.45; 38.55	32.89; 25.19; 40.59	0.75
UPDRS III	20; 13.5; 25.5	22; 16; 26	0.96
UPDRS IV	3; 2; 4	4; 1; 4	0.5

## Discussion

PD is a chronic neurodegenerative movement disorder, characterized not only by motor symptoms but also by functional, physical, and neuropsychological disabilities. DBS therapy is a well-established and effective treatment for PD, but nowadays are still controversial the sex differences in the effects of bilateral STN-DBS. Picillo et al. [4] affirm that several factors contribute this gender gap in PD. Genetic predisposition, hormonal factors, and also socio-economic influences are fundamental in the development, in the functioning of brain structures, and in defying of clinical and psychological aspects of PD patients.

The higher incidence of PD in male sex with a male/female ratio of 1/49 [15] with a delayed age onset and a slow progression in women is known [16, 17]. These conditions are, probably, ascribable to neuroprotective effect of estrogen [17–21] responsible for concentration of dopaminergic neurons in female [22] and a lower involvement of nigrostriatal fibers [16].

Motor symptoms occur later in women and manifest predominantly with reduced stiffness [23], tremor as first presenting symptom [16], increased propensity to develop postural instability, and elevated risk of levodopa-related motor complications [24–30]. In males, the disease, as demonstrated by recent studies, tends to manifest itself with the development of freezing gait [31]. Our study follows other important reports focused on gender differences of outcomes of DBS. According to Golfrè Andreasi et al. [32], there are no differences on the motor effect of STN-DBS between males and females; similar to Kim et al. [33], STN-DBS induces a similar degree of short-term and long-term effects on motor function, cognitive and depressive symptoms, and functional status between male and female PD patients. The lack of significant post-operative gender differences is most likely an effect of under-representation of women as they make up only a third of patients, so gender effects are difficult to verify. In our series, women reported more severe motor complications than men before undergoing DBS surgery. These results are similar to those of previous studies, although our cohort has been the smallest sample of patients with PD STN-DBS analyzed with respect to gender-specific findings to date [24]. Hariz et al. [34] also described an improvement in cognition and ADL following DBS specifically in women with PD; in line with these results by Hariz et al., we did not observe significant gender differences in total preoperative UPDRS I cognitive functions and in the activities of daily living (ADL) scores (UPDRS II). Regarding cognitive domains, such as memory and visuo-spatial and attention/executive skills, it has been argued that men and women can be affected in different ways, but the results are still controversial [3, 18, 35, 36]. It has been stated that women with PD might show up with a slower decline of cognitive functioning compared to men [37, 38]. In our cohort, men and women showed no significant difference (*p* < 0.01); they present the same cognitive impairment over time.

In line with literature [9, 39–44], a significant difference was found in the proportion of women undergoing surgery compered to men: women represent only 39% (*p* = 0.0257) of our cohort. We also found a longer disease duration in women before DBS (*p* = 0.04). This might be attributed to slower disease progression [16], but also to a psychological and socio-cultural choice.

Göttgens et al. [45] state that although physical disorders are similar in female and male PD patients, the psychological impact presents a “gender gap”; women suffer more from changes in their intimate relationships, while men have more difficult ties with self-presentation. So, it is legitimate to ask: is the greater weight given by the biology or by the society?

Many authors trace the tendency of women to prefer a more conservative treatment to their greater fear in facing the surgery [34, 39, 40, 46]. Hamberg et al. [47] speculate that the gender gap in DBS might be related to a greater decision-making autonomy in men as opposed to a greater need, in women, for approval by others. Although women diagnosed with PD are a sizable portion of the PD population, their specific needs are still partially underestimated. Two retrospective observational studies using Medicare and PD-MCT conducted in the USA and Germany respectively highlighted that women are less likely to have specialist care [48, 49]. Another multicentric study covering 7209 patients at 21 centers in the USA, Canada, the Netherlands, and Israel showed that women are also less likely than men to have caregiver support [50]. The reasons for this can be attributed to a longer average age of women and their natural propensity to be careers rather than recipients of care.

There are conflicting data on gender difference in the literature; however, there is one point on which there is full agreement. DBS surgery improves the quality of life significantly in both men and women. Indeed, by analyzing the postoperative UPDRS scores in both sexes, they tend to equate, showing no significant difference. Despite the worst preoperative score in UPDRS II, after 1 year of follow-up, there was no difference between men and women; therefore, women reported a greater improvement in EDL scores than in men.

This allows us to note that the advantage that women can derive from surgery is perhaps greater than that of men. Understanding the fine mechanism behind this evidence could be the key to improving treatment for both genders. Therefore, the possibility of anticipating the date of surgery in women could prevent the onset of such important subthalamic nucleus damage and obtain an even greater benefit from surgery. Increasing experimental and clinical evidence supports the idea that PD differs between women and men. Not only do men and women experience the disease differently, but different mechanisms seem to be involved in the pathogenesis of the disease. Nevertheless, we are still far away from the actual understanding of what underlies such differences. Tailored treatment counseling should take into account gender aspects in order to provide effective treatment strategies. Studies in this area are under-represented, both from the clinical and research perspectives, especially for females. Our goal is to investigate the gender gap in DBS results in a multicentric study.

## Limitation

The limitations of our study include the small size of the cohort and the short duration of the follow-up. In addition, data on the equivalent daily intake of levodopa (LEDD) have not been calculated. However, we hope this is a preliminary study of the effects of DBS by gender among PD patients. Therefore, future work with more patients is necessary to validate the results of our study.

## Conclusion

PD has a different phenotype, probably due to the intrinsic nature that distinguishes men and women. With the progression of the disease, women are at greater risk of developing highly disabling treatment-related complications, such as motor symptoms, but they have greater improvement after surgery than men. STN-DBS is a highly effective treatment for motor and non-motor symptoms of PD for both women and men but our study hints towards gender-specific outcomes in motor domains. Improving our knowledge in this field can allow us to implement strategies to identify new directions in the development of an adequate treatment of PD in terms of surgical intervention and in consideration of the gender.

## Data Availability

Due to the nature of this research, supporting data is not available nor needed.
